# Debriefing Model for Psychological Safety in Nursing Simulations: A Qualitative Study

**DOI:** 10.3390/ijerph17082826

**Published:** 2020-04-20

**Authors:** Eunjung Ko, Yun-Jung Choi

**Affiliations:** Red Cross College of Nursing, Chung-Ang University, Seoul 06974, Korea; melast06@cau.ac.kr

**Keywords:** debriefing, education, nursing, psychological safety, qualitative research, simulation

## Abstract

This study was conducted to explore nursing students’ emotional experiences during simulations, and to develop a debriefing model for psychological safety in nursing simulations by qualitatively analyzing data. Data were collected through face-to-face in-depth interviews with 23 undergraduate nursing students in South Korea. Via content analysis, nine categories were derived: fear of evaluation, burden of being observed, unfamiliarity with new ways of learning, sensitivity to interpersonal relationships, physical and emotional exhaustion, utilization of supportive relationships, decline in learning satisfaction, positive acceptance of stress, and attempts to relieve stress. On the basis of these insights, we developed the Share–Explore–Notice–Support–Extend (SENSE) debriefing model, which includes stress management and emotional support, as a strategy for effective simulation practices to reduce the negative experiences of stress in nursing students in simulation-based learning.

## 1. Introduction

There are many challenges faced by nursing education. As the number of nursing schools increase, and the admission quota expands, it is becoming increasingly difficult to access hospitals for clinical practice. In addition, most clinical practices are based on observational nursing rather than direct patient care owing to the strengthened rights of patients and growing emphasis on the importance of patient safety [1]. In response to the limitations of clinical practice, simulation-based training is drawing attention as being complementary to clinical practice in nursing education [2].

Simulation improves students’ cognitive abilities and critical-thinking skills [3,4], confidence and self-efficacy [5,6], clinical skills and clinical competency [7], and leadership skills [8]. In a study comparing the effect of simulation-based and clinical practice in maternity nursing, the satisfaction rate of simulation-based practice was higher than that of clinical practice [9].

These studies have shown simulations to be an effective teaching method, but there is a lack of evidence to make decisions regarding substituting traditional clinical experience hours with simulated experience. In a study involving 666 students from 10 nursing colleges across the United States (U.S.), three groups were randomly assigned to one of the following conditions: two years of practice with a traditional clinical practice, 25% simulated clinical practice, and 50% simulated clinical practice. The participants were evaluated for nursing knowledge, clinical competency, and NCLEX (The National Council Licensure Examination) pass rate, and no statistically significant differences were found for these three variables. Additionally, the study cohort showed no difference between the groups in manager ratings of overall clinical competence and practice readiness as a newly registered nurse during the first six months of clinical practice. These findings offered substantial evidence that up to 50% of simulations could replace existing clinical experience, and led many nursing schools to opt for up to 50% simulations instead of 100% clinical practice [10].

Despite the effectiveness of simulation learning, the perceived student stress level was higher than that in clinical practice [9]. Studies on the negative aspects of simulation training are less extensive than on the positive effects. However, for students, simulations can be synonymous with stress, and some simulation scenarios that are performed in front of others can be a source of anxiety and interfere with learning [11].

Students may be physically safe in simulation settings, but they still face risks such as failure, embarrassment, and negative evaluation from their peers or instructors [12]. Thus, a simulation situation is a learning opportunity to increase confidence or satisfaction, but it was also found to cause stress and anxiety [13]. Evaluation anxiety is also common among students because evaluating the students’ performance is an integral part of simulation learning. Evaluation anxiety is related to cognitive abilities, such as problem solving, and has a negative impact on performance [14]. In addition, instructor and peer observation while taking care of patients during simulation practice may also induce anxiety [15]. A study pointed out that simulation stress often interferes with the students’ memory of their experiences, and that learning is lost due to performance criticism [16]. Students experienced much higher stress during simulations than what the instructors expected [12]. The causes of anxiety during simulations were ranked from the highest to the lowest in the order of fear of mistakes, camera recording, teachers’ observations, and nursing performance [17].

Qualitative studies of the lived experience of nursing students in simulations reported both positive experiences, such as learning practices without fear, gaining confidence in clinical practicum, maturing in the profession, awareness and understanding, and simulation as preparation for practice [18,19,20,21]; and negative experiences, such as being anxious about having students’ mistakes exposed, anxiety from being observed, an uncertain journey to achievement and needing a navigator, and pre-experience anxiety [22,23,24,25].

There are various positive and negative experiences related to simulation practices, but studies on simulation in Korea have focused on the positive aspects. Therefore, an exploration of the negative experiences of simulation-based learning among undergraduate nursing students in Korea is needed. In addition, although simulation training has positive educational effects, undergraduate nursing students experience negative emotions in relation to simulation practice. Owing to the lack of research on this subject, it is necessary to have in-depth understanding of the experiences of nursing students’ simulation-based learning. Qualitative-research methods are often used to explore subjects’ experiences. Therefore, the objective of this study was to explore the experiences of undergraduate nursing students’ stress in simulation-based learning via a qualitative approach in order to understand their stress resulting from the simulation.

## 2. Materials and Methods

This study qualitatively analyzed data to explore and provide in-depth understanding of nursing students’ experiences of psychological safety in simulation-based learning.

Participants were undergraduate students who had completed the simulation program with more than one semester at a nursing school. Participants were recruited through advertisements posted on the bulletin boards of schools. The subjects who expressed an intention to voluntarily participate in the study were provided with explanatory documents and informed consent forms explaining the purpose of the research, the methods, and their right to withdraw. Finally, twenty-three students from four nursing schools in the Seoul, Gyeonggi, and Jeolla provinces participated.

The data were collected from May 2017 to November 2017. Each interview was conducted face-to-face in a safe place where the participants felt comfortable after informing them that it would be recorded and deleted after the study was concluded. In-depth interviews were initiated by asking the participant: “Please tell me about your experiences of stress in simulation-based learning”. Then, participants were encouraged to talk in detail about their experiences, and were drawn in by an atmosphere of constant dialog without any time limit. All interviews were recorded, and the recordings were immediately transcribed.

Data were analyzed according to the content-analysis method proposed by Graneheim and Lundman [26]. The researchers repeatedly read each line, and meaningful words, sentences, or paragraphs from the participants’ statements were selected to form meaning units. The condensed meaning units that were labeled as codes were created by summarizing the meaning units in a more general and briefer form. Among these codes, similar ones were grouped together to form subcategories, and categories were finally derived by grouping and abstracting the subcategories.

In order to ensure trustworthiness, the results of content analysis were assessed according to the qualitative-research-evaluation criteria proposed by Lincoln and Guba [27]. Two participants reviewed the codes, subcategories, and categories to ensure that the interpretations reflected their experiences, thereby enhancing the credibility of the results. Transferability was achieved by presenting the results of this study to three nursing students who had experience in simulation training, but were not participants of the study, and it was verified whether they were meaningful and applicable to them on the basis of their experiences. For auditability, two nursing-faculty members with extensive experience in qualitative research checked the entire research process and its results. Neutrality was ensured by comparing the participants’ interview contents with data from other sources to eliminate personal bias.

### Ethical Approvals

This study was conducted in accordance with the Declaration of Helsinki, and the protocol was approved by the institutional review board of the researchers’ organization (IRB no: 1041078-201701-HR-022-01). All participants voluntarily signed the consent form after the researcher explained the purpose and procedures of the study to them. It was also clearly explained that they could terminate the interview at any time; if they so desired, they could ask for suspension of their participation from the study without being disadvantaged by it in any way.

## 3. Results

Among the participants in this study, 20 students were female and 3 students were male; three students were juniors, and 20 students were seniors; 11 students were in simulation-based learning for four or more semesters, seven for three semesters, and five for two semesters. There were varying levels of the simulator: low-, mid-, and high-fidelity. All 23 participants were placed in each of these levels. Additionally, 19 students had a standardized patient experience. In the simulation practice, all 23 participants had previous experience in adult nursing, 18 students in fundamental and pediatric nursing, 15 students in maternal nursing, 11 students in psychiatric nursing, and five students in community-health nursing (Table 1).

The interview data regarding stress experienced from simulation practice were categorized, analyzed by semantic units, and grouped into similar experiences. From the interview data, 18 subcategories and nine categories were derived as factors of the students’ experience of stress arising from nursing simulations (Table 2).

### 3.1. Fear of Evaluation

The greatest source of stress reported by the participants was the grade that they would receive, and the pressure to do well because of evaluations in simulation training sessions. They needed to complete a checklist provided in the simulation training within a limited time and according to the given priorities; however, memorizing the intervention list and order within the given time is difficult. Failure to follow the checklist results in a low grade, which caused a fear of evaluation.

I’m worried about the grades I receive after the simulation, and that’s the most stressful part. Without the evaluation in the simulation practice, I will be comfortable, but making mistakes will lead to a bad grade, which makes me nervous during the simulation.

—Participant 13

In simulation training, there is a list of interventions that should be learnt within a certain time frame. For me, it is stressful to remember the order and the content of interventions according to the priority.

—Participant 16

### 3.2. Burden of Being Observed

Participants felt stressed when being watched by the instructor. Even if the instructor was not visible, it was burdensome to know that they were observing them from the control room. Additionally, it was stressful because their peers would watch the recording during the debriefing, and they were nervous about making mistakes and being embarrassed in front of their peers. Participants did not want to be recorded during the simulation training, and were dissatisfied when they watched the recorded video with their peers.

I’m very nervous because I know that the instructor is watching everything I do during the simulation. Therefore, all I can think of is to finish the practice quickly. Sometimes my classmates are watching my performance with the instructor in the control room, so I become more stressed.

—Participant 18

It is really stressful to be recorded while I do something. I would like to refuse, but I have to agree because it is used for debriefing. The worst part is that all classmates watch the recorded file together. It is so humiliating and hateful when all my mistakes are revealed in front of them.

—Participant 17

### 3.3. Unfamiliarity with New Ways of Learning

Participants experienced difficulties due to unexpected situations and sudden demands from instructors during simulation training. They were also stressed when the manikin responded unexpectedly or when they had to decide in an emergency. Because of their lack of experience in simulation on the subject, they were nervous during training. Overall, they felt unfamiliar with the new simulation learning method.

When an unexpected situation happens, I feel tensed because I have to make decisions in a short time about how to deal with it. Sometimes during a simulation, if an instructor suddenly changes the manikin’s state and it reacts unexpectedly, then I am unable to think and I just stand there because I don’t know what to do.

—Participant 8

Subjects that I have experienced a lot in simulations, such as adult nursing, seem a little easier, because I can deal with a patient’s situation or provide interventions with my previous experience. However, subjects like psychiatric or maternal nursing that are new to simulations are more difficult and stressful as the situations are unfamiliar.

—Participant 2

### 3.4. Sensitivity to Interpersonal Relationships

Participants had difficulties co-operating and communicating with team members during the simulations, and felt more that it was a simulation involving peer evaluations. Sometimes, they were also hurt by the instructor’s attitude or during the simulation, and were emotionally injured upon receiving negative feedback during the debriefing. These led participants to develop sensitivity to interpersonal relationships with team members or professors during the simulation.

I can take responsibility for the simulation by myself if I make a mistake, but in group simulations the tension does not ease because my mistake affects the overall team score. All team members practice a lot because everyone needs to cooperate, but we also fear that there may be a misunderstanding during communication.

—Participant 6

I feel bad when I meet an instructor who treats students harshly or points out a lot of mistakes during a simulation. She rarely says what I’ve done well, but only talks about mistakes, which hurts me emotionally and lowers my self-esteem. I experience a lot of stress depending on the instructor.

—Participant 9

### 3.5. Physical and Emotional Exhaustion

Participants experienced physical symptoms such as loss of appetite and sleep disturbance due to stress before or/and during the simulation, and complained of fatigue after training. They were also troubled emotionally from simulation training, and these emotional disturbances affected their performance during simulation. Overall, participants experienced physical and emotional exhaustion through simulation: “I am so worried the day before the simulation that I cannot sleep well and I lose my appetite. Because I am nervous, I sweat a lot and my hands shake during the simulation and I am so tired after I am done” (Participant 21). “I become sensitive or depressed before simulation, so I don’t want to talk to anyone. When stress increases, sometimes I get so nervous that I make such absurd mistakes during simulation that I ruin my performance” (Participant 19).

### 3.6. Utilization of Supportive Relationships

Participants relied on their team members as they prepared and collaborated as simulation teams, and utilized teamwork to relieve the stress. They were also supported by the instructor’s encouragement and guidance. In the end, participants utilized these supportive relationships and felt safe emotionally: “Simulation with the team reduces my burden. As a team, other members can make up for my weaknesses, so I am less concerned about making mistakes. I also gain knowledge by watching their performance, and relax by communicating with them” (Participant 5). “When I practice before simulation, it is helpful for my instructor to encourage me and explain the questions I ask. If I am too nervous, she says it is ok to make a mistake and her support makes me comfortable” (Participant 23).

### 3.7. Decline in Learning Satisfaction

Simulations varied according to instructors and subjects, and there was a lack of standardized education policies that made the participants feel uncomfortable. They were also disappointed because the simulated environment was not realistic, and manikins were different from humans. Finally, these led to decreased learning satisfaction: “Sometimes it is difficult to get immersed in the simulated situation because the simulator is so different from the actual patient, and it is disappointing that the scenario is not realistic. I’ve been embarrassed several times when the simulator stopped working during simulation” (Participant 22).

Each instructor or subject follows a different method of teaching and evaluation. Some give different scores to each individual, while others evaluate students as a team. Some instructors give us a guidebook in advance, but others provide verbal orientation right before the simulation starts. It won’t be so difficult, instructions were standardized.

—Participant 15

### 3.8. Positive Acceptance of Stress

Participants used textbooks and videos to build their knowledge, and practiced and prepared in advance for the simulation. They realized that practice motivated by stress was helpful for learning and gaining clinical experience. Therefore, they positively accepted the stress: “Because of stress, I practice several times in the open lab before simulation, or watch related videos and read textbooks to prepare. Without enough preparation, I get more nervous during the simulation, so I try to prepare thoroughly” (Participant 14). “In my case, it was difficult at the time due to stress, but I realized that stress was the driving force for me to practice hard and study thoroughly. It seems that preparing and gaining knowledge in advance for simulation will also help clinical practice” (Participant 6).

### 3.9. Attempts to Relieve Stress

Participants attempted to not be aware of stressful situations, and be immersed in the simulation itself, so as to not be sensitive to mistakes. They also tried a variety of methods to reduce their tension during simulation training: “I relaxed by listening to music I liked or stretching my body before simulation. Sometimes, I ate sweets like chocolate or candy to relieve my tension” (Participant 3). “I often read poetry while I prepare for simulation. I sometimes go outside the simulation room and get some fresh air because I think it is necessary to refresh my mind” (Participant 5).

I tried not to be conscious of the instructor observing my performance, or the whole process being recorded, and attempted to immerse myself in the simulation. It would be more difficult if all my classmates were watching right next to me during the simulation, but I kept thinking that this was okay.

—Participant 7

### 3.10. Conceptual Framework: Nursing Students’ Experiences of Stress in Simulation-Based Learning

**Figure 1:**
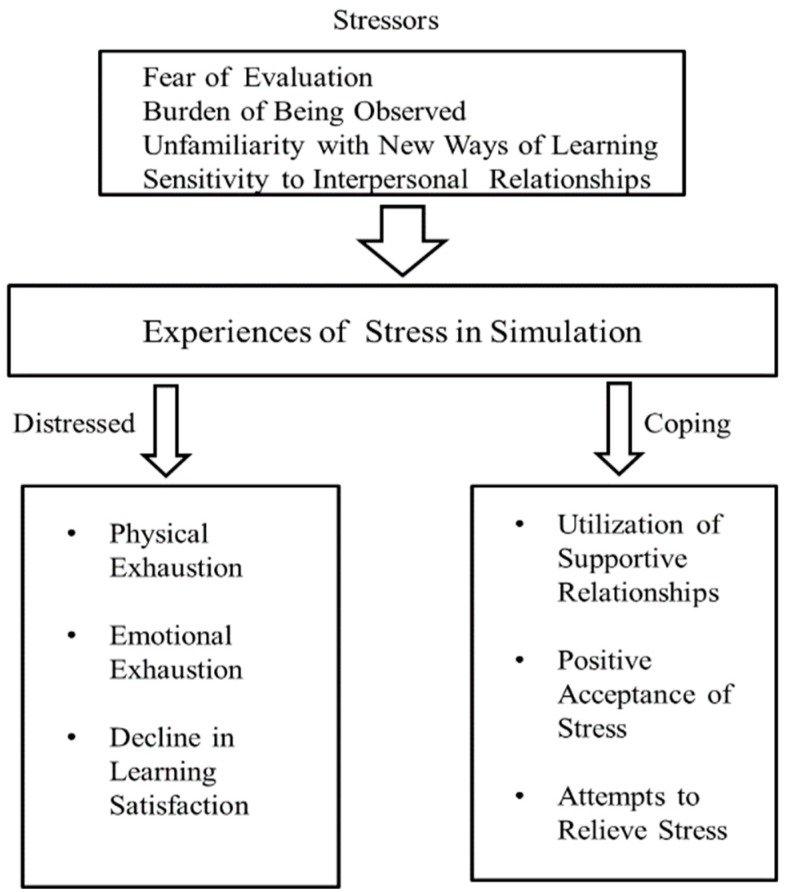
Conceptual framework: Nursing students’ experiences of stress in simulation-based learning.

The conceptual framework was derived from themes related to the experiences of stress among nursing students in simulation-based learning. Fear of evaluation, burden of being observed, unfamiliarity with new ways of learning, and sensitivity to interpersonal relationships are stressors that lead students to experience stress in simulation-based learning. These experiences of stress can cause physical and emotional exhaustion or a decline in learning satisfaction. Students can cope with stress as they utilize supportive relationships, accept stress positively, or attempt to relieve stress (Figure 1).

### 3.11. SENSE Model: Debriefing Including Emotional Support

**Figure 2:**
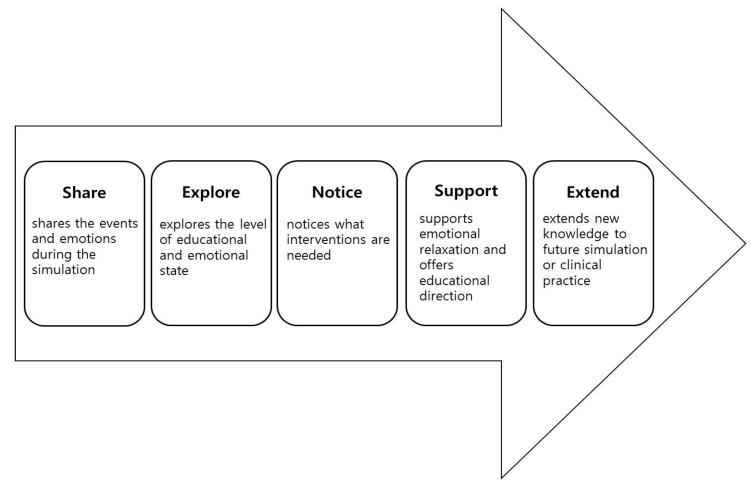
Share–Explore–Notice–Support–Extend (SENSE) model.

On the basis of categories and the conceptual framework of the present study, the Share–Explore–Notice–Support–Extend (SENSE) model was developed for stress management and emotional support, which is unlike most other debriefing models that emphasize the cognitive aspects of simulation-based learning. In the Share phase, learners share the events and emotions of the simulation practice. The instructor encourages the participants to fully disclose their feelings and the events of the simulation. The Explore phase is a step to analyze the causes of difficulty with the situation or the emotion in the simulation exercise. In the Notice phase, the instructor assesses the learner’s educational level or negative emotion, such as extreme stress or anxiety, and takes note of any help or intervention that is needed. In the Support phase, the instructor encourages students’ learning achievements, offers educational direction, and guides them through emotional relaxation therapy, such as deep breathing and maintaining a healthy lifestyle in order to reduce stress or anxiety. The Extend phase is a step to aid the students in extensively applying their newfound knowledge and experience in future simulation exercises and clinical practice (Figure 2).

## 4. Discussion

The results of this study indicated that participants experienced stress in simulation training and felt pressured when being evaluated. This is consistent with findings that anxiety increases when there is a formal summative evaluation after the simulation training [16]. It is also consistent with another study that showed that students experienced stress when they exceeded the stipulated time limit or rushed to complete the simulation [12]. Taking this into consideration, the instructor should not excessively increase the weight of the grades in simulation-education sessions or prevent the grades of simulation from greatly affecting other courses. It is important to ensure that the purpose of the simulation is not the grade but the learning.

Participants experienced stress due to being watched by others, especially due to the instructor observing what they performed. It was also burdensome to have their peers observing them. These factors are conceptually similar and related to previous findings [12,17]. Therefore, allowing other students to observe performance may unnecessarily cause anxiety in the participants, and it may hinder immersion in the simulation, so instructors should avoid it. In addition, it is necessary to seek the participants’ understanding about the recording for the debriefing. In the debriefing, recorded files should be played for each individual team, rather than for the whole class.

Participants had difficulties coping with unexpected situations during the simulation. These difficulties were even worse in the junior than in the senior year. Although the number of participants in these two years was not the same, the difference may be because coping skills and clinical performance improve as the student progresses in the course, and their simulation experience increases. The high level of anxiety among junior students who had no clinical experience, and the positive correlation between clinical performance and anxiety during simulation are consistent with previous studies [28,29]. Therefore, it is necessary to provide basic information about a simulation scenario, even if the scenario is not disclosed for the purpose of learning. In addition, the instructor should plan simulation training so that the experience of the participants can be expanded. Even if an unexpected situation occurs, students should be equipped with sufficient knowledge and skills in advance so that they can solve it by themselves. Participants experienced difficulty in their relationship with their team members and the attitudes of the instructor as they practiced the simulation. This is consistent with worries about damaging teamwork, one of the results of simulation experience of nursing students in a previous qualitative study [22]. It is necessary to appropriately distribute team-based and individual training in accordance with the purpose of training when planning simulation training, because it may interfere with the learning due to the burden of the relationship with peers. In addition, the instructor needs to use supportive language and be wary of authoritarian attitudes to avoid hurting students’ feelings during the debriefing.

These are various ways in which participants experienced stress related to the simulation. They also positively accepted stress and acquiesced that it may be helpful in learning. However, they reported experiencing physical, emotional, and cognitive difficulties because of the stress. In this study, female students complained of more physical and emotional symptoms due to stress or anxiety compared to males. Similarly, in a study conducted in Hong Kong, female nursing students reported greater symptoms of anxiety than male students [30]. However, the number of male participants was too small, so there is a need to investigate larger samples in future studies.

In the future, when planning simulation training, such interventions should be developed that reduce negative experiences due to stress. The purpose of a debriefing session is to give students positive feedback after identifying the stressful emotional responses of the students after simulation training, and to give them an opportunity to identify the knowledge and skills they need to reflect on their behavior [31]. Further, debriefing is considered to be one of the most important elements for emotional coping after simulation training. Effective debriefing maximizes the opportunity of learning and helps emotional release [32].

According to the results of this study, a number of stressors led nursing students to experience stress in simulation-based learning. Even though some students can positively cope with this stress, others may be physically or emotionally distressed, and these experiences can cause a decline in learning satisfaction. Therefore, it is necessary to develop interventions in order to substantially reduce these negative experiences through emotional support when planning simulation education. In summary, debriefing is an important component of the simulation training, and it is also effective in providing emotional support to students who have experienced stress from the simulation practice in debriefing. Therefore, it is necessary to ensure emotional support to relieve stress during debriefings.

The SENSE model includes emotional support in nursing simulation based on the emotional factors of nursing students. Through the whole process of debriefing, the learners share emotions, and the instructors analyze the difficulties, assess the level of stress, notice needed interventions for the learners, and guide emotional relaxation. One of the most widely used debriefing models is the Gather–Analyze–Summarize (GAS) model proposed by the American Heart Association. In the Gather phase, the instructor listens to students’ thoughts and perspectives on behaviors after the simulation exercise, and collects the information. In the Analyze phase, contents are analyzed. The Summarize phase is a step for reviewing and confirming the contents learned through the educational session [33]. The debriefing model of the National League for Nursing (NLN) can be found in NLN’s online learning center, Simulation Innovation Resource Center (SIRC). It has three stages: introduction, middle analysis, and summary, and it is useful in the development of nursing curricula [34].

Although there are various types of debriefing models, as described above, it is necessary to introduce the SENSE model including emotional support into nursing simulations in order to reduce the negative impact of stress from simulation-based learning on the emotions of nursing students and the uniqueness of nursing education. The SENSE model, the debriefing tool for stress management, and emotional support suggested in this study, requires verification of their effectiveness; therefore, follow-up studies must be conducted.

Overall, this study has some limitations. First, it was conducted only for nursing students in Korea and, therefore, cannot be generalized. Second, this was a qualitative study; further quantitative studies need to be conducted on this topic.

## 5. Conclusions

This study was a qualitative study applying in-depth interviews as a tool to analyze the contents of psychological safety of undergraduate nursing students in simulation training. The main findings of this study concerned stressors of nursing students in simulation training, their experiences, and the results of stress. These findings provide basic data of the experience of stress in simulation training among undergraduate nursing students. This helps in the development of an intervention to reduce nursing students’ negative experiences in future simulation-training plans. These can also be used as a tool for the development of interventions, such as the SENSE model, which is a debriefing tool for stress management. They can help reduce the negative consequences of these stressors through the provision of emotional support in future simulation-exercise planning.

## Figures and Tables

**Table 1:** General participant characteristics (*N* = 23).

Variables	*N*
**Gender**	
Male	3
Female	20
**Grade**	
Junior	3
Senior	20
**Number of completed semesters of simulation training**	
Two semesters	5
Three semesters	7
Four or more semesters	11
Type of simulator in simulation training *	
Low-fidelity simulator	23
Mid-fidelity simulator	23
High-fidelity simulator	23
Standardized patient	19
Course of simulation training *	
Fundamental nursing	18
Adult nursing	23
Maternal nursing	15
Pediatric nursing	18
Psychiatric nursing	11
Community-health nursing	5

Note: * Multiple answers possible.

**Table 2:** Content analysis: nursing students’ experiences of stress in simulation-based learning.

Categories	Subcategories
Fear of evaluation	Worrying about grades Having difficulty complying with the criteria
Burden of being observed	Being distressed by performing in front of others Objecting to be recorded
Unfamiliarity with new ways of learning	Being embarrassed by unexpected situations Feeling nervous because of the lack of experience and knowledge
Sensitivity to interpersonal relationships	Facing difficulty working with team members Being hurt by the instructor
Physical and emotional exhaustion	Being troubled physically Being troubled emotionally
Utilization of supportive relationships	Utilizing teamwork Being supported by the instructor
Decline in learning satisfaction	Facing difficulty in an unstandardized method of teaching Feeling disappointed with the simulation-based learning
Positive acceptance of stress	Preparing beforehand Experiencing positive effects of stress on learning
Attempts to relieve stress	Trying not to be conscious of stressful situations Trying to reduce tension
